# Supplementary material for: A new QRS detector stress test combining temporal jitter and F-score (JF) reveals significant performance differences amongst popular detectors
Source: PLoS One. 2024 Nov 11;19(11):e0309739. doi: 10.1371/journal.pone.0309739 (PMC11554035; doi:10.1371/journal.pone.0309739)
Supplement: Attachment — Submitted filename: rebuttal.pdf [file pone.0309739.s001.pdf]

PONE-D-23-23819

Bernd Porr & Peter W Macfarlane

We thank all reviewers for the very constructive feedback.

Reviewer 1

Key terms of equations must be defined.
We have explained all key terms. In particular we explained "argmin", "for all" and "card" which are central for both the sensitivity and JF calculation.

Give more details about the Dataset. Also, the authors have specified the reasons for selecting the dataset.
We have added a section explaining why we are using this dataset. There are multiple reasons: sample precision annotation, different degrees of signal to noise ratio (from sitting to jogging) and two different QRS morphologies.

The authors should mention to where the proposed system is applicable.
Indeed, this was missing. We have added this to the Introduction and also at the end of the discussion.

The references should be the last five years.
We are sorry we cannot accept this demand as for example Pan and Tompkins 1985 is integral to a discussion on this topic.

Reviewer 2

Computation of accuracy depends on the knowledge of the true negative (TN), namely (equation 15): Accuracy = (TP + TN) / (TP + TN + FP + FN) However, in the context of QRS detection, the concept of true negatives is not applicable. The authors currently use an alternative definition of the accuracy which does not include the true negative. However, these alternative definitions do not conform to the standard definitions provided in the literature, and their interpretation is unclear. Therefore, this indicator should be removed from the manuscript or rearranged.

We thank the reviewer for this suggestion. Indeed, TN was only approximated and needed to be removed. Instead, we use now the F1-score, now commonly used in the literature. This has also an impact on the name of our measure which we changed from JA to JF.

I recommend rearranging the manuscript in relation to the research steps, for a better fluency of the information presented. Thus, overall, the structure of the paper is difficult to follow in relation to the dedicated sections.
We have re-arranged the manuscript to increase fluency and also added additional paragraphs to set the scene for the steps ahead.

The proposed methodology design should be more clarified. The presentation of the database and of the performance measures for detection efficacy should be presented in experimental setup.
This also relates to the above comments and we agree that sections had to be re-arranged. We are a bit confused what the reviewer means with "experimental setup" but we agree that the way the Methods section was ordered was confusing and the new ordering should fix this. We have also added more context justifying the creation of the new database, namely, that it offers different SNR levels, two different QRS morphologies and more precise annotation than the MITDB. We have also added a plot showing an example trace from the MITDB illustrating that the problem with this data base is annotation jitter.

Table 1. The description of the table suggests that all results presented are from the GUDB database, whereas the first column contains results obtained from the MIT-BIH AR database, which is not mentioned in any way and is misleading. Furthermore, what is meant by the phrase unspecified tolerance (%). In the following columns, it is specified that these are results obtained (I think) from the work on the basis of signals from GUDB records. However, combining the results in this way may mislead readers. Another inaccuracy is the value given after the plus/minus sign. What is this value? How was it determined?
We thank the reviewer for this valuable feedback. Firstly there was no paragraph describing the table overall and we have added this. This now explains the different columns and also tells the reader that columns 3 and 4 are averages and standard deviations based on 3911 QRS complexes. We have added what we mean with "unspecified tolerance". We have added that we have obtained the new sensitivity and JF readings from our new GUDB. These velues also match the plots in Figs 6 and 7.

Table 1 of Hamilton's paper is assigned the wrong bibliographic number, 31 instead of 24
Many thanks. That error has now been corrected.

The Discussions should be oriented towards summarizing the results and comparing them with those of similar studies and presenting the advantages and novelty of the proposed algorithm. In addition, the limits of the research carried out should be identified and briefly presented. Nevertheless, are important to mention the perspectives for the application in clinical practice of the new algorithm, and new research directions derived from the research carried out.
We agree that a comparison of our JF performance measure with similar studies was missing from the discussion as was the use of different ECG databases in other studies compared to our approach of having created a database with realistic noise. These shortcomings have now been corrected. We also stress that this paper is NOT about new QRS detection algorithms but how to benchmark them. We have also added to the Discussion the impact of this approach on clinical practice, current limitations and the way forward.

There is no conclusion section.
For Plos one a conclusion is optional and thus we have not written one.

No translation of abbreviations, e.g. EMG.
We have added translations of abbreviations.

What is the relevance to the manuscript of the information that the Pan&Tompkins algorithm was originally written for the Z80 microprocessor?
Thank you for this comment. We agree that this was an irrelevant detail and have removed it.

There is a lot of formulations in the paper that are not supported by the results (e.g. line 353-354).
Thanks for pointing out that especially references to Table 1 had a few mistakes. We have double checked and all formulations now support the results.

No description of the numerical experiment in which a standard deviation value of 35.8% is obtained (line 367). How many times were the calculations performed?
This was added at the top when the table with the stats was introduced. In total, there were 3911 QRS complexes throughout the study and used for all detectors. This means that 3911 estimates for our JF performance measure and for the traditional sensitivity measure were available for calculation of mean and SD.

It seems to me that for the quality of the work as a whole, it would be advisable to use the MIT-BIH AR database and then compare the results. By which the work would be undoubtedly better appreciated.

We have outlined in the Introduction the flaws of the MIT-BIH AR database and the problems with other databases. None of the MIT databases offers increasing noise levels, all have inaccuracies in the annotations and do not contain different morphologies. We feel that we have now clearly justified not using the MIT DB.

The paper says that the annotation of heartbeats in ECG signals was performed with a Python script using an interactive plot. Were the determined QRS positions verified by an expert cardiologist? From the information available, it appears that the development of this software ended in 2018/2019 and furthermore, no information is provided about the signal processing used.
We have explained the annotation procedure in detail now in the methods section.
The database was created and recorded in 2018/2019. However, the software is actively maintained and has been revised for this revision of the paper. There are in total 3 citations in the paper referring to both dataset and python scripts: dataset, detector code and JF performance measure. We created first the dataset, then the different detectors and finally the performance measure. This has been an effort which lasted over a couple of years.
The raw ECG recordings have not undergone any signal processing and were directly stored. Upon retrieval of the data both 0.1Hz highpass filtering and 50Hz notch filtering was applied. We have added this to the MS.

Reviewer 3

The contribution of the paper needs to be properly specified in the abstract.
We have rewritten the abstract that it reflects its contribution.

The figures should be place with in the text for better readability of the article during review process.
We are sorry but the Plos guidelines dictate that graphical material has to be provided separately.

What are the key technical problems that this paper tries to solve and what are the drawbacks of the existing QRS detectors?
The paper is NOT about the drawbacks of the existing detectors but how to benchmark them. The problem is that almost all papers report very high sensitivity values which makes a comparison between sensors pointless. Our new benchmark JF in conjunction with the new dataset corrects this shortcoming.

In the introduction, I suggest that the author rearrange the introduction section.

This is a very valid point raised by all 3 reviewers. We have re-arranged the introduction and expanded it to make clear the nature of the problem this paper addresses. See above.

The paper needs to discuss recent developed QRS detection methods and include in the comparison. Some of the recent methods are in the following works: Design of A Biorthogonal Wavelet Transform Based R-Peak Detection and Data Compression Scheme for Implantable Cardiac Pacemaker Systems Efficient QRS complex detection algorithm based on Fast Fourier Transform Design of Efficient Fractional Operator for ECG Signal Detection in Implantable Cardiac Pacemaker Systems Hardware Emulation of a Biorthogonal Wavelet Transform Based Heart Rate Monitoring system
This paper is NOT about detectors but about benchmarking them. Having said that, we have added a paragraph referencing wavelet based detectors and acknowledged that there are newer developments in wave detection.

The availability of dataset should be provided in the paper before reference section via link.
We have added a link in the text.

Why post processing is required.
That subheading is a misnomer and we now call this section more appropriately: "Annotation procedure".

Is the annotation was done through software or by medical practitioners.
We have made this clear that it was first done by an algorithm and then corrected by an experienced engineer.

Discuss the computational complexity of proposed model and compare with the existing methods.
We believe there is no need to compare the computational complexity as this paper is about a *benchmark measure* which runs offline and not about optimising a realtime detector. See also our replies above.

There are lots of typo error and grammatical error. That should be corrected.
We have proof read the MS now numerous times and believe we have addressed all typos and grammatical errors.
